# Self-Esteem as a Mediating Mechanism in the Association Between Emotional Experiences and Academic Performance in School Students: The Moderating Role of Age

**DOI:** 10.3390/ejihpe16090123

**Published:** 2026-08-25

**Authors:** Roxana Bernal-Rojas, David Quispe-Sanca, Gutember Peralta-Eugenio, Wilter C. Morales-García, Liset Z. Sairitupa-Sanchez

**Affiliations:** 1 Postgraduate School, Universidad Peruana Unión, Lima 15102, Peru; 2 Facultad de Ciencias de la Salud, Escuela Profesional de Psicología, Universidad César Vallejo, Chimbote 02710, Peru; gperaltae@ucvvirtual.edu.pe; 3 Facultad de Teología, Universidad Peruana Unión, Lima 15102, Peru; wiltermorales@upeu.edu.pe; 4 Escuela Profesional de Psicología, Universidad Señor de Sipán, Chiclayo 14001, Peru

**Keywords:** self-esteem, emotions, academic performance, school students, adolescence

## Abstract

*Background*: Academic performance does not depend solely on cognitive abilities, but also depends on socioemotional processes. Emotional experiences may be associated with school performance; however, their effects are often inconsistent, suggesting the need to examine mediating mechanisms such as self-esteem and developmental conditions such as age. *Objective*: We aimed to analyze the association between positive and negative emotional experiences and academic performance in school students, assessing the mediating role of self-esteem and the moderating effect of age. *Methods*: A cross-sectional explanatory study was conducted with 325 students from Lima, Peru (149 boys and 176 girls). Emotional experiences were assessed using the PANAS-N, self-esteem with the Rosenberg Self-Esteem Scale, and academic performance with final grades in Communication and Mathematics. Data were analyzed using partial least squares structural equation modeling (PLS-SEM), with bootstrapping based on 10,000 subsamples. *Results*: Positive emotional experiences were positively associated with self-esteem (*β* = 0.434, *p* < 0.001) and academic performance (*β* = 0.272, *p* < 0.001). Negative emotional experiences were negatively associated with self-esteem (*β* = −0.489, *p* < 0.001) but showed no significant direct association with academic performance (*β* = −0.005, *p* > 0.05). Self-esteem was positively associated with academic performance (*β* = 0.114, *p* = 0.043). The model explained 40.8% of the variance in self-esteem and 11.3% of the variance in academic performance. Age moderated only the association between negative emotional experiences and self-esteem (Δ*β* = 0.183, *p* = 0.035). *Conclusions*: Self-esteem plays a significant mediating role in the association between emotional experiences and academic performance, and this effect is especially relevant during adolescence. The findings suggest that self-worth constitutes a key psychological process for understanding how emotional experiences are linked to school performance.

## 1. Introduction

Academic performance constitutes a strategic indicator of school adjustment and human development, as it reflects not only the degree to which educational demands are met but also broader processes of cognitive, motivational, and psychosocial adaptation. Its deterioration has been linked to risk trajectories that include early school dropout, lower life satisfaction, a greater propensity for substance use, and antisocial behavior; in contrast, its strengthening is associated with better opportunities for educational mobility and with cumulative contributions to human capital and countries’ economic development (Carroza-Pacheco et al., 2025; Chue & Lim, 2024). In addition, recent evidence suggests that school performance may also be compromised by adverse early experiences, conflictive school climates, and peer victimization, including bullying, all of which simultaneously affect psychological well-being, self-esteem, and school engagement (Figueiredo, 2024; Qu et al., 2024). Consequently, identifying the mechanisms that explain differences in academic performance remains a priority objective in psychological and educational research.

The contemporary literature has moved beyond exclusively cognitive explanations of academic achievement and recognizes that it emerges from the interaction of psychological, social, and contextual factors operating simultaneously within the student’s school experience (Carroza-Pacheco et al., 2025; Chue & Lim, 2024; Newar et al., 2025). This perspective is especially relevant in school-aged populations because learning does not occur in isolation, but rather within diverse institutional, familial, and sociocultural contexts that shape participation, sense of belonging, self-perception, and emotional responses to academic demands (Morlà-Folch et al., 2022). Within this framework, emotional experiences have gained particular prominence, especially in late childhood and adolescence, developmental stages characterized by heightened affective reactivity, greater sensitivity to social and academic evaluation, and still-maturing processes of emotional self-regulation and executive control (Liu et al., 2025; R. Wu & Yu, 2022; Yang et al., 2025). This interest is not incidental: emotions do not merely accompany learning but shape the way students attend to, interpret, anticipate, cope with, and persist in the face of the demands of the school context.

From a conceptual perspective, emotional experiences comprise affective states that integrate subjective, cognitive, motivational, expressive, and physiological components and that can generally be organized around positive and negative valences (Pekrun, 2024; Pekrun et al., 2017; Sandín, 2003). At the same time, using Lazarus and Folkman’s transactional model, emotions can be understood as responses derived from cognitive appraisal processes through which individuals interpret whether a situation represents harm, threat, challenge, or benefit, as well as whether they have sufficient resources to cope with it (Lazarus & Folkman, 1984). In the school setting, this implies that the same academic situation—for example, a test or a class presentation—may generate different emotional reactions depending on the student’s subjective appraisal and perceived control. In education, many of these affective responses are expressed as achievement emotions or academic emotions, that is, emotions linked to learning activities, evaluations, outcomes, and performance expectations (Pekrun, 2024; Qi et al., 2025). In that sense, although this study uses the term emotional experiences, it is conceptually situated within the field of academic emotions, since it refers to affective states experienced in relation to school tasks, demands, and outcomes. This distinction is theoretically relevant because it shifts the analysis from a nonspecific understanding of affect to a situated conceptualization functionally related to school demands and to the meanings students assign to those demands.

The most robust explanatory framework for understanding these associations is the Control-Value Theory (CVT), which posits that academic emotions arise from students’ subjective appraisals of perceived control over the activity or outcome, as well as the value that they attribute to that academic experience (Pekrun, 2006). CVT proposes that emotions differ not only in terms of valence (positive vs. negative) but also in terms of activation level (activating vs. deactivating) and focus (activity-oriented or outcome-oriented), which helps explain why nominally “positive” emotions do not always lead to adaptive outcomes and why some “negative” emotions may, under certain conditions, be associated with greater behavioral mobilization (Pekrun, 2024; Yang et al., 2025). Thus, positive-valence emotions may include experiences such as enjoyment, hope, pride, or relief, whereas negative-valence emotions typically include anxiety, shame, boredom, frustration, or hopelessness. However, these should not be treated as isolated categories, nor should their effects be assumed to depend solely on whether they are pleasant or unpleasant, because their influence is also determined by their intensity, duration, focus, and regulatory function in academic behavior. Functionally, emotions such as enjoyment or pride tend to promote engagement, persistence, and self-regulation, whereas anxiety, boredom, or hopelessness tend to impair attention, cognitive efficiency, and sustained effort (Pekrun, 2024; Pekrun et al., 2017; Qi et al., 2025). Likewise, the study of academic emotions requires a broad and multicultural perspective, since their frequency, intensity, and meaning may vary depending on the school context, social norms, interactions with peers and teachers, and the opportunities for participation provided by the school (Morlà-Folch et al., 2022).

Indeed, recent reviews indicate that the relationship between emotional experiences and academic performance shows inconsistencies in direction, magnitude, and statistical significance. Some studies report that certain positive emotions—such as relief, pride, or relaxation—may be associated with less favorable performance; conversely, some negative emotions, such as anxiety or frustration, may relate to better outcomes in specific contexts (R. Wu & Yu, 2022). Likewise, non-significant associations have been documented for emotions such as enjoyment and boredom (Yang et al., 2025). This pattern suggests that the effect of emotions on performance does not depend solely on their valence but also on intermediate psychological processes that may amplify, channel, or attenuate their impact on academic behavior. At this point, the inclusion of mediating variables becomes essential in order to refine the theoretical explanation of the phenomenon. Within this framework, incorporating a mediating variable requires specifying what type of psychological process might translate emotional experiences into performance differences. The present study proposes that such experiences influence not only the immediate execution of tasks but also the global evaluation students construct about themselves from repeated experiences of success, failure, social comparison, feedback, and acceptance or rejection within the school context. From this perspective, self-esteem constitutes a theoretically plausible mechanism insofar as it may capture how the accumulated affective load of the school experience affects students’ self-valuation and, from there, their academic adjustment.

Among the potentially involved mechanisms, self-esteem holds a particularly relevant position. Self-esteem can be defined as the global evaluation individuals make of their own personal worth, integrating judgments of competence, dignity, and self-acceptance (León García, 2024; B. Zhao et al., 2025). In the school context, this construct serves not only a self-evaluative function but also a regulatory one, insofar as it influences the interpretation of experiences, expectations of coping, tolerance for error, and persistence in the face of difficulty. High levels of self-esteem are typically associated with greater confidence, perseverance, and school adjustment, whereas low levels are related to insecurity, avoidance, emotional vulnerability, and poorer performance (Rey Manzano, 2024; Z. Shi et al., 2023). In addition, self-esteem is particularly sensitive to adverse interpersonal and school experiences; for example, victimization, peer rejection, and other forms of negative social exposure may undermine self-worth and affect school adjustment, especially during adolescence (Figueiredo, 2024). Converging evidence has likewise shown that emotional experiences, particularly negative ones, may erode self-esteem (X. Wu et al., 2021), and that lower self-esteem may compromise academic engagement and learning (Rey Manzano, 2024). When it is stated that there is converging evidence for this link, this refers not only to statistical associations between negative affect and low self-esteem but also to observable behavioral and school-related consequences such as withdrawal, reduced participation, adjustment difficulties, interpersonal conflict, and greater vulnerability to bullying or exclusion, all of which may amplify the deterioration of academic performance (Figueiredo, 2024). Despite this, evidence specifically examining self-esteem as a mediating mechanism between emotional experiences and academic performance in school students remains limited. This gap is substantial because it prevents a clear understanding of why similar affective configurations do not necessarily lead to equivalent consequences for performance. In particular, evidence simultaneously examining the mediating role of self-esteem and the moderating effect of age in the association between emotional experiences and academic performance in school populations remains scarce, especially in Latin American contexts.

Moreover, the relevance of academic performance as a dependent variable lies not only in its frequent use in educational research but also in the fact that it functions as a synthetic outcome of the interaction between personal resources—such as self-esteem, motivation, and emotional regulation—and contextual conditions—such as school climate, interpersonal relationships, family involvement, and educational opportunities (Figueiredo, 2024; Morlà-Folch et al., 2022; Qu et al., 2024). This clarification makes it possible to understand that academic performance is not an exclusively cognitive product, but rather an observable expression of broader adaptive processes that unfold within the school experience.

Added to this complexity is the potentially differentiating role of age. Developmental change significantly alters the stability of self-esteem, the intensity of emotional responses, and the sophistication of self-regulatory processes. Longitudinal evidence shows that self-esteem tends to be more fluctuating during adolescence and more stable in later developmental stages (Orth & Robins, 2014). At the same time, the maturation of executive functions and emotional control systems transforms the way children and adolescents process feedback, academic demands, and experiences of success or failure (Casey, 2015; Roller & Steinberg, 2020). Likewise, the literature indicates that the frequency, intensity, and meaning of academic emotions are not uniform across children and adolescents, but rather change over the course of development and also as a function of contextual and sociocultural conditions. During the transition into adolescence, self-consciousness, social comparison, sensitivity to external judgment, and vulnerability to negative school experiences tend to intensify, which may cause negative emotions to affect self-esteem more strongly (R. Wu & Yu, 2022). this regard, it has been reported that negative emotions may affect self-esteem more intensely in adolescents than in children, and that the frequency and intensity of academic emotions vary according to age, in addition to other contextual and sociocultural factors (R. Wu & Yu, 2022; Xu, 2024). Complementarily, recent meta-analytic evidence has shown that adverse experiences in childhood and adolescence are associated with poorer performance indicators, grade repetition, and a greater need for educational support, reinforcing the importance of examining how emotional vulnerability and self-valuation may interact with age to influence academic performance (Qu et al., 2024). However, the moderating role of age in the relationships among emotional experiences, self-esteem, and academic performance remains insufficiently examined, especially in school populations.

Taken together, these antecedents suggest that the relationship between emotions and academic performance should be analyzed through more integrative models capable of incorporating both intermediate psychological mechanisms and developmental conditions that modulate the strength of these associations. Within this framework, the present study aims to examine the association between positive and negative emotional experiences and academic performance in school students, assessing the mediating role of self-esteem and the moderating effect of age. Although academic performance was estimated from specific school subjects, the study focuses on a general socioemotional mechanism linked to students’ self-valuation within their school experience. More specifically, the study is situated in school students from educational institutions in Lima, Peru, and seeks to offer a developmentally and contextually sensitive explanation, avoiding the assumption that the relationships among emotion, self-esteem, and performance operate uniformly across all age groups or school populations. From a theoretical perspective, this study expands the applicability of Control-Value Theory by incorporating a self-evaluative variable as an explanatory mechanism in the relationship between emotion and performance. From an applied perspective, its findings may guide future socioemotional support initiatives in the school setting, particularly with regard to students’ self-valuation in response to academic demands. Consequently, this study seeks to provide a more precise and developmentally sensitive explanatory model for understanding academic performance in school students.

### 1.1. Literature Review

#### 1.1.1. Self-Esteem

Self-esteem has been conceptualized as a global evaluation of the self, referring to the subjective appraisal that individuals make of their own worth, dignity, and personal acceptance (León García, 2024; B. Zhao et al., 2025). It is a self-referential construct that integrates cognitions, affects, and accumulated experiences and plays a central role in the organization of psychological and social adjustment. Although its study has a long tradition, disagreements remain regarding its conceptual boundaries, partly due to its proximity to related constructs such as self-concept, self-image, and self-perception, which, while related, should not be assumed to be equivalent without theoretical precision (Escrivá-Martínez et al., 2025).

Structurally, Rosenberg’s classical formulation conceptualizes self-esteem as a relatively stable global evaluation; however, later approaches have suggested that, during certain stages of development, this evaluation may manifest through specific domains, such as competence, social acceptance, or physical appearance (Escrivá-Martínez et al., 2025). In school populations, self-esteem is particularly relevant because it shapes how students interpret success and failure, cope with academic demands, and regulate their persistence when facing challenging tasks. High self-esteem is associated with greater confidence, perseverance, and adaptive coping, whereas low self-esteem is typically linked to emotional vulnerability, doubts about one’s own worth, and lower academic persistence (Rey Manzano, 2024; Z. Shi et al., 2023). In addition, self-esteem can be undermined by repeated exposure to adverse school experiences, including peer rejection, relational conflict, and victimization, highlighting its sensitivity to the school environment and its potential role as an explanatory mechanism between emotional experience and academic performance (Figueiredo, 2024). Therefore, its inclusion as a mediating variable in models of academic performance is supported both theoretically and empirically.

#### 1.1.2. Emotional Experiences

Emotional experiences refer to the set of affective states that individuals experience in response to meaningful situations and encompass subjective, cognitive, motivational, expressive, and physiological responses (Pekrun, 2006; Pekrun et al., 2017). From the perspective of Lazarus and Folkman, these responses depend not only on the event itself but also on the cognitive appraisal individuals make of the situation and of their own coping resources, which is especially useful for understanding the diversity of emotional reactions that may emerge in response to the same school-related demand (Lazarus & Folkman, 1984). These experiences can be broadly grouped into positive affect and negative affect (Sandín, 2003), or identified through specific emotions such as anxiety, shame, frustration, sadness, pride, relief, hope, or relaxation (Simonič & Osewska, 2023; R. Wu & Yu, 2022). However, within educational contexts, a more precise approach requires considering their functionally situated nature.

In this regard, achievement emotions constitute a subcategory of particular interest because they are directly linked to learning activities, performance, evaluation, and academic outcomes (Pekrun, 2006, 2024; Qi et al., 2025). These emotions not only accompany the school experience but also influence the selection of strategies, attentional focus, effort mobilization, and persistence. Control-Value Theory (CVT) proposes that their effects depend on the interaction between valence, activation, and focus, implying that the same emotion may serve different functions depending on the context in which it arises and the meanings that sustain it (Pekrun, 2024). For this reason, positive and negative valence should not be treated as a merely descriptive classification: in school practice, positive emotions such as pride or relief are not always adaptive, and negative emotions such as anxiety may, in certain contexts, mobilize preparation or effort. In addition, academic emotional experience must be understood within specific sociocultural contexts, since the ways emotions are expressed, interpreted, and regulated may vary among students depending on school organization, pedagogical relationships, and the classroom social climate (Morlà-Folch et al., 2022). Therefore, the analysis of emotional experiences in education requires a process-oriented rather than merely descriptive reading.

#### 1.1.3. Academic Performance

Academic performance refers to the level of achievement attained by students as a result of their participation in formal learning processes. It is commonly operationalized through objective indicators such as grades or assessment outcomes, although theoretically it can also be understood as a specific dimension of learning performance, which integrates cognitive, behavioral, and affective components (Qi et al., 2025). From this perspective, academic performance represents the observable expression of multiple underlying processes, including self-regulatory capacity, persistence, engagement, and adaptation to the demands of the school environment.

Its relevance as a dependent variable in educational research lies in the fact that it functions as a synthetic outcome of the interaction between personal resources and contextual conditions. In particular, performance may be influenced by school climate, the quality of relationships with peers and teachers, family involvement, and the presence or absence of conditions of exclusion and school violence; therefore, its study requires an integrative approach that connects the psychological with the social and contextual (Morlà-Folch et al., 2022; Qu et al., 2024). Consequently, studying its determinants requires explanatory models that take into account both structural variables and psychological mechanisms closely related to academic behavior (Newar et al., 2025).

#### 1.1.4. Association Between Emotional Experiences and Academic Performance

Empirical research has consistently shown that emotional experiences are associated with academic performance; however, the direction and magnitude of this association are not uniform. In general, positive emotions tend to be related to greater attention, cooperation, interest, engagement, and persistence in tasks, which in turn promotes better learning outcomes (Liu et al., 2025; Rana et al., 2025; R. Wu & Yu, 2022). Nevertheless, certain positive emotions—such as relief or relaxation—may be linked to reduced sustained effort or a deactivation of academic behavior, producing less favorable associations with performance (R. Wu & Yu, 2022).

Similarly, although negative emotions are typically associated with impaired attention, cognitive interference, and reduced efficiency in problem solving, some findings suggest that not all negative emotions operate in a uniformly dysfunctional manner. In certain contexts, emotions such as anxiety or frustration may mobilize control behaviors, preparation, or increased effort, resulting in positive associations with performance (R. Wu & Yu, 2022). Likewise, non-significant effects have been reported for emotions such as enjoyment and boredom (Yang et al., 2025). These findings indicate that the relationship between emotion and performance is shaped by intermediate processes that have not yet been sufficiently specified in school populations. In addition, recent evidence suggests that the impact of these experiences may intensify when students are exposed to adverse developmental conditions, such as negative early experiences or exclusionary school dynamics, which further underscores the need to study intermediate psychological mechanisms such as self-esteem (Figueiredo, 2024; Qu et al., 2024).

#### 1.1.5. Self-Esteem as a Mediating Mechanism

The plausibility of self-esteem as a mediator is supported by its dual function: on one hand, it acts as an outcome of emotional experience; on the other, it shapes how students engage with academic demands. Repeated emotional experiences—especially negative ones—can alter the global evaluation students make of themselves, eroding their perceptions of competence, confidence, and coping capacity (X. Wu et al., 2021). In turn, weakened self-esteem may negatively affect perseverance, engagement, willingness to exert effort, and the interpretation of academic outcomes, thereby generating an indirect impact on performance (Rey Manzano, 2024; Z. Shi et al., 2023).

From a mediation perspective, this pattern suggests that part of the link between emotional experiences and academic performance could be explained by changes in students’ self-valuation. This interpretation is reinforced by studies showing that exposure to negative relational experiences, such as bullying, victimization, or social rejection, may damage self-esteem and, through it, compromise school adjustment and academic participation (Figueiredo, 2024). Although the methodological literature has established robust criteria for assessing indirect effects (X. Zhao et al., 2010), substantive evidence examining this process in school students remains limited. Therefore, modeling self-esteem as a mediating mechanism is not only theoretically consistent but also necessary for a more precise understanding of the heterogeneity observed in the effects of emotions on performance.

#### 1.1.6. Age as a Moderating Condition

Age constitutes a developmental condition that may alter both the stability of self-esteem and the intensity and functionality of emotional responses. Adolescence is characterized by a reorganization of cognitive, affective, and social processes that increases sensitivity to external judgment, comparative self-evaluation, and emotional variability, which may intensify the effects of emotional experiences on self-referential variables such as self-esteem (Orth & Robins, 2014; Roller & Steinberg, 2020). At the same time, the progressive maturation of executive functions modifies the ability to regulate impulses, reinterpret experiences, and sustain coping strategies when facing academic demands (Casey, 2015).

The available evidence suggests that negative emotions may affect self-esteem more strongly in older groups within the school-age period and that academic emotional experiences are not distributed uniformly across development (R. Wu & Yu, 2022; Xu, 2024). Moreover, this heterogeneity is explained not only by chronological age but also by the social, academic, and relational demands characteristic of each school stage, making the distinction between children and adolescents conceptually necessary. The recent literature also shows that adverse experiences during childhood and adolescence are associated with poorer school outcomes overall, suggesting that age may modify the way emotional vulnerability, self-esteem, and performance are articulated throughout the school trajectory (Qu et al., 2024). Consequently, age may not only accompany but also modify the strength of the associations among emotional experiences, self-esteem, and academic performance. Evaluating this moderating effect therefore allows for the introduction of a developmentally sensitive reading into the proposed theoretical model.

**Figure 1:**
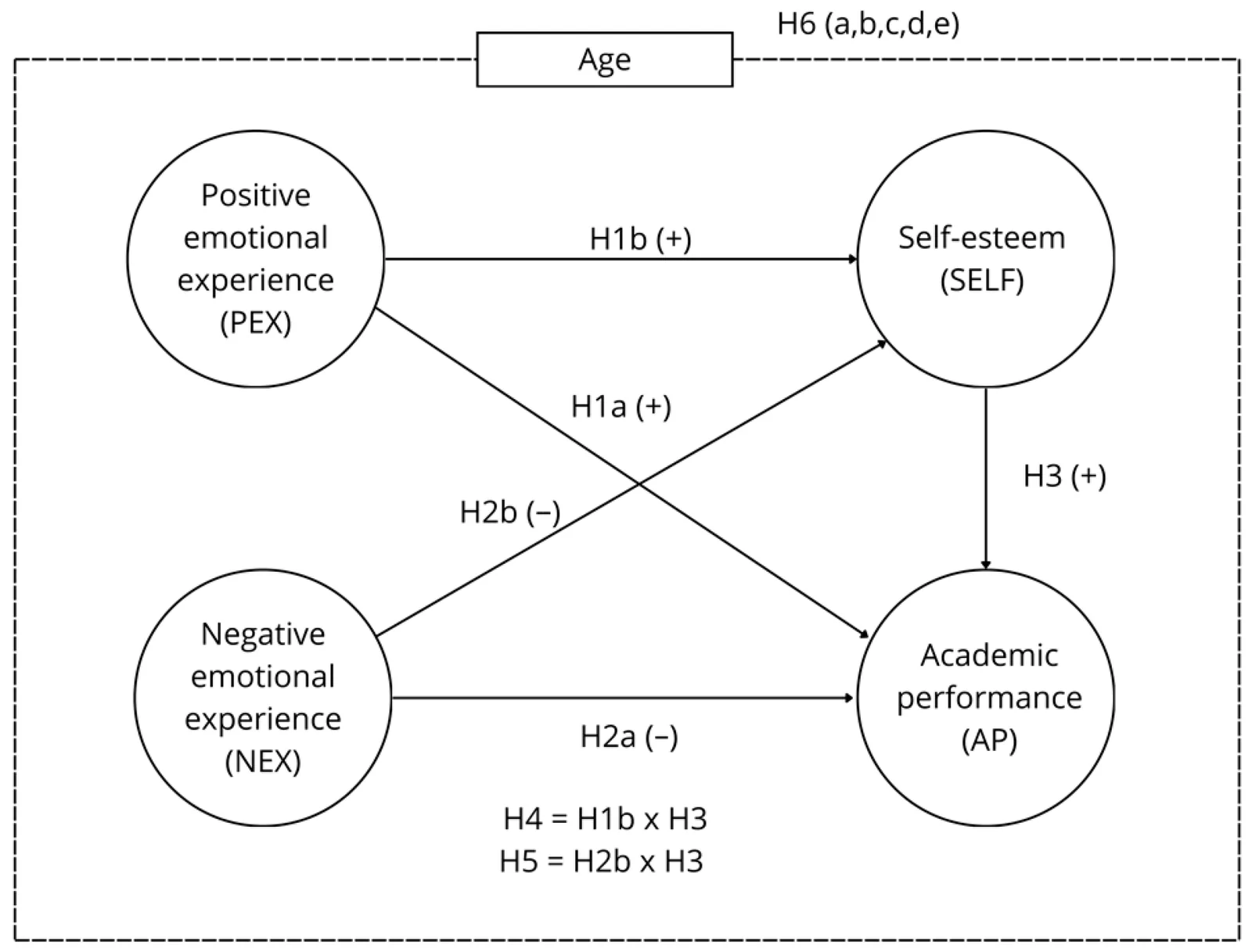
Theorical model.

Based on the theoretical and empirical framework presented, the following hypotheses are proposed (Figure 1):

**H1a.**  *Positive emotional experiences are positively and significantly associated with higher levels of academic performance.*

**H1b.**  *Positive emotional experiences are positively and significantly associated with self-esteem.*

**H2a.**  *Negative emotional experiences are negatively and significantly associated with academic performance.*

**H2b.**  *Negative emotional experiences are negatively and significantly associated with self-esteem.*

**H3.**  *Self-esteem is positively and significantly associated with academic performance.*

**H4.**  *Self-esteem has a significant indirect effect on the association between positive emotional experiences and academic performance.*

**H5.**  *Self-esteem has a significant indirect effect on the association between negative emotional experiences and academic performance.*

**H6a.**  *Age moderates the association between positive emotional experiences and the level of academic performance.*

**H6b.**  *Age moderates the association between negative emotional experiences and the level of academic performance.*

**H6c.**  *Age moderates the association between positive emotional experiences and self-esteem.*

**H6d.**  *Age moderates the association between negative emotional experiences and self-esteem.*

**H6e.**  *Age moderates the association between self-esteem and the level of academic performance.*

## 2. Materials and Methods

### 2.1. Study Design and Participants

A quantitative, non-experimental, cross-sectional study with an explanatory scope was conducted to examine the relationships among latent variables using partial least squares structural equation modeling (PLS-SEM). This approach was considered appropriate because it allows the simultaneous estimation of the measurement model and the structural model in explanatory research involving latent psychological constructs. The final sample consisted of 325 school students from educational institutions in Lima, Peru, of whom 149 were male and 176 were female. Participants were selected through non-probabilistic convenience sampling, a strategy suitable in educational contexts where access depends on institutional availability and authorization from participating schools (Otzen & Manterola, 2017). Prior to data collection, the minimum required sample size was estimated using Soper’s online calculator, yielding a minimum requirement of 119 participants, assuming an effect size of *λ* = 0.30, statistical power of 1 − *β* = 0.80, a significance level of α = 0.05, and 31 predictors specified in the analytical model. Consequently, the observed sample size exceeded the minimum requirement for the proposed analysis. The inclusion criteria were (a) being enrolled in an educational institution during the 2025 academic year, (b) being of Peruvian nationality, and (c) having informed consent from parents or legal guardians, as well as student assent when applicable. Participants with incomplete questionnaires and those who reported a history of psychiatric disorders, suicidal ideation, or severe learning difficulties were excluded in order to reduce potential confounding factors. For comparative analyses, participants were classified into two age groups: primary school students aged 9 to 12 years (*n* = 139) and secondary school students aged 13 to 17 years (*n* = 186).

### 2.2. Measures

Emotional Experiences: Emotional experiences were assessed using the Positive and Negative Affect Scale for Children and Adolescents (PANAS-N), developed by Sandín (2003). The Spanish version was used, consisting of 20 items with prior evidence of adequate internal consistency (α = 0.80) in Spanish-speaking child and adolescent populations (Padros-Blazquez et al., 2023). The instrument includes two dimensions: positive affect (10 items) and negative affect (10 items). Responses are recorded on a five-point Likert scale ranging from 1 = Not at all to 5 = Very much. Higher scores indicate greater intensity or frequency of the corresponding emotional experience.

Self-Esteem: Self-esteem was measured using the Rosenberg Self-Esteem Scale (RSES) (Rosenberg, 2015). The Spanish version validated by Atienza et al. (2000) was employed, consisting of 10 items with reported adequate reliability (α = 0.80). The instrument has a unidimensional structure and uses a five-point Likert scale ranging from 1 = Strongly disagree to 5 = Strongly agree. Higher scores reflect higher levels of global self-esteem.

Academic Performance: Academic performance was operationalized on the basis of the students’ official final averages in the subjects of Communication and Mathematics, according to institutional assessment records. For the analysis, the mean of both grades was used as an indicator of overall academic performance in core curricular areas. This decision was based on the fact that both subjects are core curricular domains and are widely used as benchmarks of general school performance in the Peruvian educational context; however, it is acknowledged that institutional grades may be influenced by evaluation criteria specific to each school and teacher. Therefore, this indicator should be interpreted as an approximation of overall academic performance rather than as an exhaustive measure of school achievement.

### 2.3. Procedure

Data collection was conducted using a self-report technique through a paper-based questionnaire that integrated the scales included in the study. The administration took place in group sessions previously coordinated with the participating educational institutions.

Before the instruments were administered, standardized instructions were provided regarding the general purpose of the study, the response procedure, and the confidential nature of the information. During the administration, the research team remained present to clarify any comprehension questions while ensuring that participants’ responses were not influenced. The estimated time to complete the questionnaire was 40 min.

Subsequently, the official academic records corresponding to the subjects of Communication and Mathematics were collected and integrated into the database for joint analysis with the psychological variables assessed.

### 2.4. Statistical Analysis

Data analysis was conducted in several stages. First, the potential presence of common method bias was assessed using Harman’s single-factor test in order to examine whether a substantial proportion of the observed variance could be attributed to the shared measurement source (Awais et al., 2023; Fuller et al., 2016; Podsakoff et al., 2003). Second, descriptive statistics were calculated for the study variables, including mean, standard deviation, skewness, and kurtosis, to characterize the distribution of the data and evaluate univariate normality according to criteria suggested in the methodological literature (Bandalos & Finney, 2018; Kline, 2016).

The main analysis was conducted using partial least squares structural equation modeling (PLS-SEM) with SmartPLS 4 (version 4.1.1.4). In the first stage, the measurement model was evaluated by examining external factor loadings, internal consistency, convergent validity through average variance extracted (AVE), and discriminant validity, following methodological guidelines recommended for PLS-SEM-based models (Hair et al., 2011; Henseler et al., 2015).

In the second stage, the structural model was examined by estimating path coefficients and their statistical significance through bootstrapping with 10,000 subsamples. Additionally, the explained variance (*R*^2^) of the endogenous variables and effect sizes (*f*^2^) were evaluated according to criteria reported in the specialized literature (Hair et al., 2019; Khalilzadeh & Tasci, 2017; Zinbarg et al., 2005). The mediating effect of self-esteem was examined through the analysis of indirect effects, following the procedure proposed by Nitzl et al. (2016).

Because the study included comparisons between age groups, measurement invariance was verified prior to the multigroup analysis using the MICOM procedure. Specifically, the following factors were sequentially evaluated, namely (a) configural invariance, (b) compositional invariance, and (c) equality of means and variances, following the recommendations of Henseler et al. (2015). Once invariance at the required level was established, multigroup analysis (PLS-MGA) was conducted using bootstrapping to test potential differences in structural coefficients between age groups (Sarstedt et al., 2011).

### 2.5. Ethical Considerations

The study was conducted in accordance with ethical principles applicable to research involving human participants and minors. Participation was voluntary, anonymous, and confidential. Prior to data collection, informed consent was obtained from parents or legal guardians, along with student assent when applicable. The research protocol was reviewed and approved by the Research Ethics Committee of the Graduate School of Universidad Peruana Unión, under approval number 2025-CE-EPG-00030. All collected information was used exclusively for academic and research purposes.

## 3. Results

### 3.1. Descriptive Statistics and Common Method Bias

The descriptive statistics showed that the variables included in the study presented skewness and kurtosis values within acceptable ranges for assuming univariate normality, according to the criteria reported in the methodological literature (Bandalos & Finney, 2018; Kline, 2016) (Table 1). Both in the age groups analyzed separately and in the total sample, the observed distributions did not show severe deviations from normality. In addition, the possible presence of common method bias was assessed using Harman’s single-factor test. The results indicated that the variance explained by a single factor was 26%, a value below the critical threshold of 50% suggested in the literature (Fuller et al., 2016), no relevant evidence of common method bias attributable to the use of self-report measures was identified.

### 3.2. Measurement Model Evaluation

The evaluation of the measurement model showed that most indicators presented acceptable factor loadings. In the final model, 19 items showed loadings above 0.70, while five items presented loadings between 0.50 and 0.69. According to the recommendations of Hair et al. (2019), the latter may be retained when their inclusion contributes to the overall adequacy of the construct and does not compromise reliability or convergent validity. Of the 31 items initially considered, six were removed in order to optimize the psychometric fit of the model. Specifically, item au06 from the self-esteem scale, item em12 from the positive emotional experience dimension, and items em04, em06, em13, and em15 from the negative emotional experience dimension were eliminated. Regarding internal consistency, all constructs showed adequate values of Cronbach’s alpha and composite reliability (*CR*), with coefficients above 0.70. Likewise, convergent validity was satisfactory, as all latent constructs presented average variance extracted (*AVE*) values greater than 0.50, meeting the criteria established in the methodological literature (Hair et al., 2013) (Table 2).

### 3.3. Discriminant Validity

Discriminant validity was evaluated using the HTMT (heterotrait–monotrait ratio) criterion. The results showed that all HTMT indices were below the recommended threshold of 0.85, supporting adequate discrimination among the constructs included in the model (Henseler et al., 2015; Q. Shi et al., 2025). Consequently, the findings suggest that the latent variables assessed represent conceptually distinct domains (Table 3).

### 3.4. Structural Model Evaluation

The results of the structural model indicated that four of the five direct-effect hypotheses were empirically supported (Table 4). In particular, positive emotional experience showed a positive and significant association with self-esteem (*β* = 0.434, *p* < 0.001) and academic performance (*β* = 0.272, *p* < 0.001). In turn, negative emotional experience showed a negative and significant association with self-esteem (*β* = −0.489, *p* < 0.001) but did not exhibit a significant relationship with academic performance (*β* = −0.005, *p* > 0.05). Finally, self-esteem was positively and significantly associated with academic performance (*β* = 0.114, *p* = 0.043). In terms of explanatory power, positive and negative emotional experiences jointly explained 40.8% of the variance in self-esteem (*R*^2^ = 0.408). Furthermore, the model explained 11.3% of the variance in academic performance (*R*^2^ = 0.113), considering the combined contribution of emotional experiences and self-esteem. Regarding effect sizes, the relationships between emotional experiences and self-esteem showed relatively larger magnitudes than those observed for academic performance. In contrast, the effect of self-esteem on academic performance was smaller in magnitude, suggesting a more modest, though statistically significant, contribution.

### 3.5. Evaluation of the Mediating Effect

**Figure 2:**
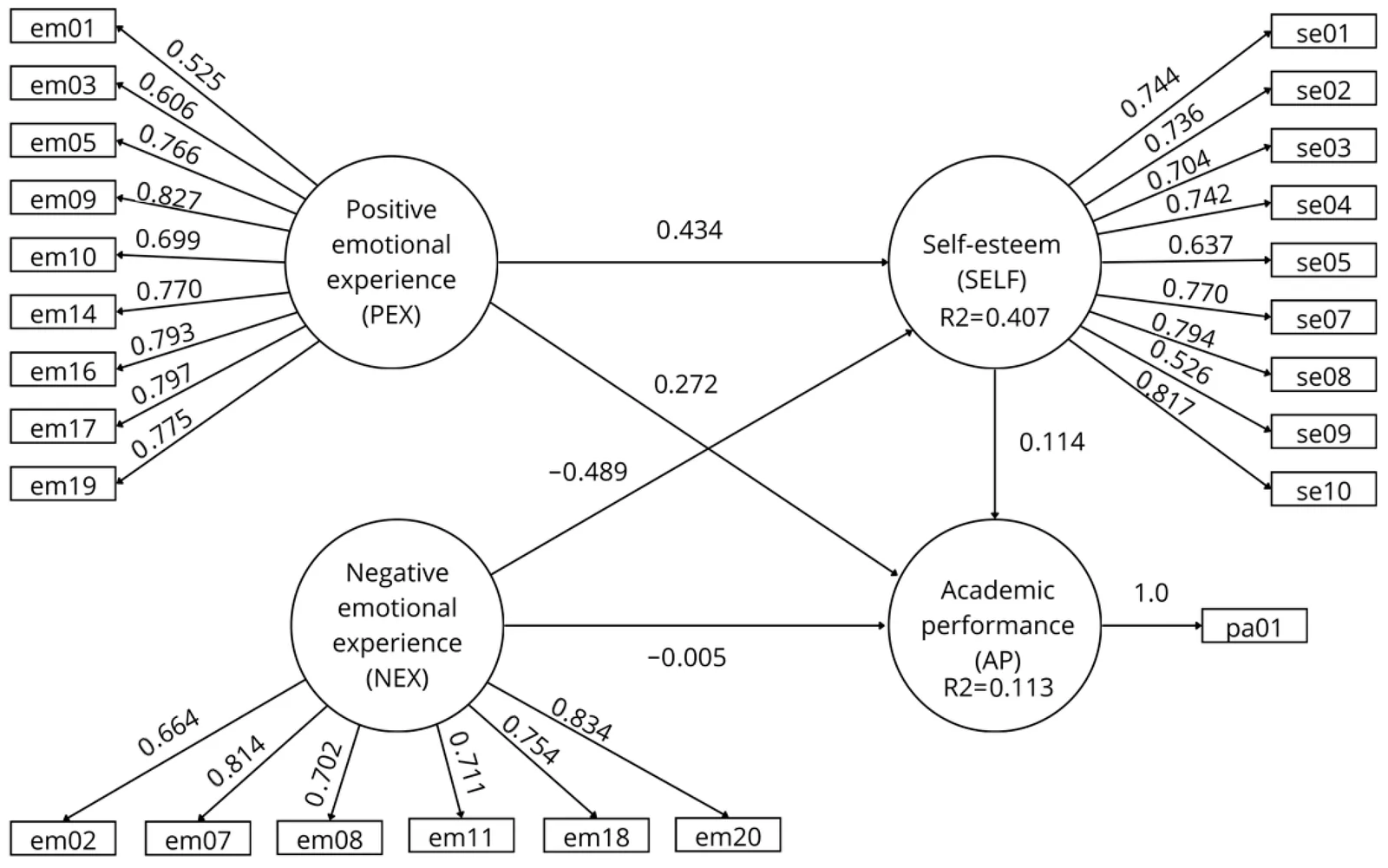
Final Structural Model. Note. PEX = positive emotional experience; NEX = negative emotional experience; SELF = self-esteem; AP = academic performance.

The mediation analysis showed that self-esteem played a significant role in the association between emotional experiences and academic performance (Table 5; Figure 2).

In the case of positive emotional experience, both the direct effect on academic performance (*β* = 0.272, *p* < 0.001) and the indirect effect mediated by self-esteem (*β* = 0.049, *p* = 0.027) were statistically significant. According to the criteria proposed by X. Zhao et al. (2010) and Nitzl et al. (2016), this pattern corresponds to complementary partial mediation, since both the direct and indirect effects were significant and operated in the same direction. The value of *VAF* = 15.27% suggests that a limited, though significant, proportion of the total effect was channeled through self-esteem.

In contrast, for negative emotional experience, the direct effect on academic performance was not significant, whereas the indirect effect through self-esteem was significant (*β* = −0.056, *p* = 0.026). This pattern is consistent with indirect-only mediation, indicating that the influence of negative emotional experience on academic performance was expressed primarily through its association with self-esteem. The *VAF* = 93.33% supports this interpretation.

### 3.6. Measurement Invariance and Multigroup Analysis

The evaluation of measurement invariance using the MICOM procedure first showed that configural invariance was established, indicating that the model structure was equivalent across the two age groups. Second, compositional invariance was established, as the original correlations of the constructs were higher than the corresponding 5th percentile, allowing partial invariance between groups to be assumed (Table 6). However, in the third step of the MICOM procedure, full equality of means and variances was not observed for all constructs, and therefore full invariance was not achieved. Nevertheless, because partial invariance was established, it was methodologically appropriate to proceed with the multigroup comparison of structural coefficients.

Once partial invariance was confirmed, multigroup analysis (PLS-MGA) was conducted to compare structural coefficients between students aged 9–12 and those aged 13–17. The results showed that only one path presented statistically significant differences between groups: the association between negative emotional experience and self-esteem (H2b/H6d) (Table 7).

Specifically, this relationship was significantly stronger in the 13–17 age group (*β* = −0.549) than in the 9–12 age group (*β* = −0.366), with a coefficient difference of Δ*β* = 0.183, *p* = 0.035. In contrast, the remaining paths in the model showed differences between groups, but these differences did not reach statistical significance.

### 3.7. Summary of Findings

Overall, the results indicate that positive emotional experiences were favorably associated with both self-esteem and academic performance, whereas negative emotional experiences were negatively related to self-esteem but not directly to academic performance. Self-esteem, in turn, showed a positive association with academic performance and acted as a significant mediating mechanism in both emotional pathways. Furthermore, the multigroup analysis suggests that age significantly moderated only the relationship between negative emotional experience and self-esteem, indicating that the impact of negative emotions on self-evaluation was stronger among older students.

## 4. Discussion

The present study aimed to examine the mediating role of self-esteem in the association between emotional experiences and academic performance in school students, as well as to evaluate the potential moderating effect of age.

Although studies that simultaneously analyze emotional experiences, self-esteem, and academic performance within a mediation model remain limited, the results of the present study converge with previous research that has identified self-esteem as a central psychological mechanism in academic functioning. In particular, the pattern observed for negative emotional experiences is consistent with studies in which self-esteem has also operated as a mediating variable between indicators of psychological distress and academic performance, such as research on the impostor phenomenon (Newar et al., 2025). Although this phenomenon does not constitute an emotion in the strict sense (Pekrun, 2006; Sandín, 2003; Simonič & Osewska, 2023), it is closely related to negative affective states such as insecurity, self-doubt, and fear, which allows for conceptual convergence with the findings reported here. Regarding direct effects, the results showed that negative emotional experiences were negatively and significantly associated with self-esteem, consistent with prior evidence in school populations (Liu et al., 2025; Newar et al., 2025; X. Wu et al., 2021). Similarly, positive emotional experiences were positively associated with self-esteem, and self-esteem, in turn, was positively related to academic performance, which aligns with previous studies involving student populations (Liu et al., 2025; Muñoz Alcívar et al., 2025; Newar et al., 2025). In this sense, the observed empirical pattern supports the analytical assumptions underlying the existence of a mediating effect (X. Zhao et al., 2010). One of the most relevant contributions of this study is that it allows a reinterpretation of the relationship between negative emotional experiences and academic performance. In the analyzed model, these experiences did not show a significant direct association with academic performance; instead, their relationship with performance was expressed indirectly through self-esteem. This finding provides greater precision to the theoretical debate regarding whether negative emotions affect academic performance positively, negatively, or ambiguously (Rana et al., 2025; Thomas & Wulff, 2024; R. Wu & Yu, 2022). Rather than assuming a direct and uniform effect, the results suggest that the impact of negative emotional experiences depends on intermediate psychological processes, among which self-esteem occupies a central role. This interpretation is consistent with the literature indicating that higher self-esteem promotes confidence, perseverance, and adaptive coping with academic challenges, whereas lower levels of self-esteem are associated with greater vulnerability and poorer academic performance (Rey Manzano, 2024; Z. Shi et al., 2023). From an applied perspective, these results suggest that it is insufficient to focus interventions solely on reducing the presence of negative emotional experiences in the classroom. Instead, it appears particularly important to strengthen the psychological resources that buffer their effects, especially self-esteem. In other words, the problem may not lie exclusively in the emergence of negative emotions, but rather in how these emotions affect students’ self-evaluation and, through it, their academic performance. In the case of positive emotional experiences, the observed complementary partial mediation indicates that self-esteem does not replace their effect on academic performance but rather amplifies it. This means that positive emotional experiences are directly associated with better performance and, additionally, contribute to academic outcomes by strengthening self-esteem. In this sense, the findings reinforce the idea that positive emotions constitute a favorable resource for learning and that their effects may be amplified when accompanied by a strong sense of self-worth. At the same time, these results suggest that some inconsistencies reported in the literature, for example, when emotions such as relief, pride, or relaxation are linked to less favorable outcomes (R. Wu & Yu, 2022), may depend on the intervention of other psychological or contextual variables not included in the present model. In the Peruvian context, this clarification is especially relevant, since Mathematics and Communication are core curricular areas within the National Curriculum for Basic Education and form part of the competencies that the educational system monitors as a priority in national learning achievement assessments. In this sense, performance derived from both areas does not represent an arbitrary indicator, but rather an expression of school performance in domains considered fundamental to students’ educational trajectories in the country. Nevertheless, because the psychological variables assessed in this study correspond to general constructs, the findings should be interpreted as evidence of the relationship between broad socioemotional processes and performance in key academic areas of the Peruvian school context, rather than as specific effects attributable to any one subject in particular (Currículo Nacional de La Educación Básica, 2016; Minedu, 2016).

The multigroup analysis showed that the model achieved partial invariance, which allowed structural paths to be compared between the two age groups. However, the moderation hypotheses were not generally supported. Of the five relationships evaluated, only one showed statistically significant differences between groups: the association between negative emotional experiences and self-esteem. Specifically, this relationship was significantly stronger in the group of students aged 13 to 17 than in the group aged 9 to 12, suggesting that older adolescents show greater vulnerability of self-esteem when facing negative emotional experiences. This finding is consistent with the developmental literature identifying adolescence as a stage characterized by greater instability in self-esteem and heightened sensitivity to internal and external factors affecting self-evaluation (Dybro Liengaard, 2024; Orth & Robins, 2014; Roller & Steinberg, 2020; R. Wu & Yu, 2022; Xu, 2024). Complementarily, adolescence has also been documented as a developmental period particularly susceptible to contextual, social, and emotional influences, which may intensify the impact of adverse experiences on psychological functioning (Guo et al., 2024). Within this framework, the results suggest that the transition into adolescence may represent a critical period in which negative emotional experiences have a greater potential to undermine self-esteem. This finding is particularly relevant because it identifies a specific point of vulnerability within the model, rather than a generalized moderation effect across all structural paths. On the other hand, the fact that the remaining structural relationships did not show significant differences between groups suggests that many of the central associations within the model remain relatively stable across children and adolescents. This indicates that, although age introduces heterogeneity in the relationship between negative emotions and self-esteem, it does not substantially alter the other associations examined. At the same time, this pattern suggests that other developmental, familial, or contextual variables may be moderating those relationships for which no significant differences were observed in the present study.

### 4.1. Implications

From a theoretical standpoint, this study contributes to expanding the understanding of Pekrun’s Control-Value Theory by incorporating self-esteem as a relevant explanatory mechanism in the association between emotional experiences and academic performance. The findings suggest that the relationship between emotion and performance should not be interpreted exclusively in direct terms but also interpreted through self-evaluative processes that influence the way students confront school demands. In this sense, self-esteem emerges as a key psychological variable for understanding why similar emotional experiences may be associated with different levels of academic performance.

Likewise, the study provides evidence that age plays a specific rather than generalized moderating role within the model. In particular, it significantly moderates only the relationship between negative emotions and self-esteem, whereas the rest of the associations remained relatively stable across the age groups evaluated. This finding reinforces the need to incorporate a developmental perspective into the analysis of academic and emotional adjustment during basic education.

From a practical perspective, the results suggest that academic performance depends not only on cognitive variables but also on socioemotional processes, and that self-esteem may constitute a relevant psychological resource within these dynamics. Consequently, the findings may help guide future socioemotional support strategies in the school setting, although their applied implications should be interpreted with caution, given that the study did not assess variables specific to the academic context nor allow for the establishment of causal relationships.

The previous literature supports the usefulness of programs aimed at socioemotional well-being and the strengthening of personal resources associated with school adjustment (Acosta-Román et al., 2024; Xu, 2024). In line with this, the findings of the present study suggest that educational institutions might consider self-esteem as a potential focus of attention within broader preventive and psychoeducational efforts. However, more research incorporating variables sensitive to the academic context is needed before more precise intervention recommendations can be derived for specific curricular areas.

In particular, the results indicate that adolescents aged 13 to 17 showed greater vulnerability in the relationship between negative emotional experiences and self-esteem compared with children aged 9 to 12. This finding suggests the value of considering differentiated socioemotional support according to age group, especially during developmental stages characterized by greater sensitivity to social comparison, external feedback, and self-evaluation. Nevertheless, this implication should be understood as a preliminary direction rather than as direct evidence regarding the effectiveness of specific interventions.

### 4.2. Limitations and Future Research

Despite its contributions, the present study has several limitations that should be considered when interpreting the findings. First, the cross-sectional design prevents causal relationships from being established conclusively, as it only allows associations among variables to be examined at a specific point in time. Although the PLS-SEM model makes it possible to evaluate structural relationships consistent with a theoretical framework, these relationships should be interpreted with caution and not as definitive evidence of causality.

Likewise, one limitation of the study is that the psychological variables were assessed using general instruments, whereas academic performance was derived from specific areas of the school context, particularly Mathematics and Communication. Although the model showed adequate results within this sample, the findings should be interpreted as associations between general socioemotional processes and school performance within this particular analytical context, rather than as subject-specific relationships. In this regard, future research should incorporate measures that are more sensitive to the academic domain in order to examine whether these associations are replicated at the curricular-area level.

Second, the sample was limited to students from a specific region of Peru, which restricts the generalizability of the results to other geographic, cultural, and socioeducational contexts. It is possible that contextual characteristics, such as norms governing emotional expression, family expectations regarding performance, teachers’ feedback styles, forms of social comparison among peers, or patterns of school coexistence, influence the emotional and academic dynamics observed. For this reason, future studies should expand the sample to other regions of the country and even to other Latin American contexts in order to test the stability of the proposed model across different sociocultural settings.

Third, although the model explained a substantial proportion of the variance in self-esteem, its explanatory power for academic performance was more modest. This suggests the presence of other relevant factors not included in the model. In this sense, future studies could incorporate additional variables such as social support, socioeconomic status, family climate, family stress, academic self-efficacy, teacher support, or self-confidence, with the aim of building more comprehensive models.

Future research should prioritize longitudinal or prospective designs to clarify the temporal ordering of the relationships proposed in this study. Such designs would make it possible to examine whether emotional experiences predict subsequent changes in self-esteem and academic performance, whether self-esteem functions as a stable mediating mechanism over time, and how these associations vary across developmental stages. In addition, repeated assessments would allow researchers to capture intraindividual changes in emotional experiences and self-worth during the transition from childhood to adolescence, providing stronger evidence for developmentally sensitive explanatory models of academic functioning.

## 5. Conclusions

The findings of the present study suggest that self-esteem constitutes a significant mediator in the association between emotional experiences and academic performance among students in Peru’s basic education system. In particular, self-esteem showed complementary partial mediation in the relationship between positive emotional experiences and academic performance, indicating that these emotions are directly associated with better performance and, additionally, reinforce that effect through higher self-esteem. In other words, self-esteem does not replace the effect of positive emotions but rather helps strengthen it.

On the other hand, self-esteem showed exclusive indirect mediation in the relationship between negative emotional experiences and academic performance. This means that negative emotional experiences were not directly and significantly associated with academic performance but were indirectly associated with it through their relationship with self-esteem. Consequently, the results suggest that the impact of negative emotions on school performance depends, to a large extent, on how they affect students’ self-worth.

Taken together, these findings underscore the relevance of psychological health and self-evaluative processes in understanding academic success. In this sense, the results suggest that self-esteem could represent a relevant focus within future socioemotional support strategies in the school setting. Nevertheless, this implication should be interpreted with caution, given that the study did not assess variables specific to the academic context and does not allow causal conclusions to be drawn regarding the effectiveness of concrete interventions. Consequently, a comprehensive educational approach that takes students’ psychological well-being into account may be relevant for addressing the challenges of the school context.

With regard to age moderation, it is concluded that age showed significant differences only in the association between negative emotional experiences and self-esteem. In particular, adolescents aged 13 to 17 showed greater vulnerability in self-esteem in response to negative emotional experiences than children aged 9 to 12. In contrast, the rest of the model’s structural relationships remained relatively homogeneous across both groups. This finding suggests the value of considering differentiated socioemotional support according to developmental stage, especially during adolescence, a period in which self-worth appears to be more sensitive to negative emotional experiences.

## Figures and Tables

**Table 1:** Descriptive statistics of the study variables categorized by age group and total sample (*N* = 325).

Variable	Group 1 (9 to 12 Years, *n* = 139)				Group 2 (13 to 17 Years, *n* = 186)				Total Group (*N* = 325)			
	*M*	*SD*	*g* ^1^	*g* ^2^	*M*	*SD*	*g* ^1^	*g* ^2^	*M*	*SD*	*g* ^1^	*g* ^2^
PEX	2.955	1.211	0.003	−0.82	3.769	1.098	−0.78	0.254	3.026	1.178	−0.02	−0.73
NEX	4.316	0.849	−1.29	1.503	3.226	1.085	−0.24	−0.39	4.148	0.991	−1.17	0.913
SELF	2.205	0.756	0.919	0.545	2.514	0.789	0.446	−0.14	2.374	0.8	0.646	−0.04

Note. PEX = positive emotional experience; NEX = negative emotional experience; SELF = self-esteem; *M* = mean; *SD* = standard deviation; *g*^1^ = skewness; *g*^2^ = kurtosis.

**Table 2:** Factor Loadings, Internal Consistency, and Convergent Validity of the Measurement Model (*N* = 325).

Construct	Item	Code	Loading	Alpha	*CR*	*AVE*
Self-Esteem (SELF)	I almost always feel good about myself	au01	0.744	0.884	0.907	0.524
	Sometimes I think I’m useless *	au02	0.736			
	I believe I have some good qualities	au03	0.704			
	I almost always think I’m a failure *	au04	0.742			
	I am capable of doing things as well as others	au05	0.637			
	I don’t think I have much to be proud of *	au06 **	—			
	I have a positive attitude toward myself	au07	0.77			
	I have a positive attitude toward myself *	au08	0.794			
	I feel as valuable as everyone else	au09	0.526			
	I would like to have more respect for myself *	au10	0.817			
Positive Emotional Experience (PEX)	I am interested in people or things	em01	0.525	0.892	0.912	0.54
	I am a lively person; I tend to get excited	em03	0.606			
	I feel like I have vitality or energy	em05	0.766			
	I get excited (about things, people, etc.)	em09	0.827			
	I feel proud (of something), satisfied	em10	0.699			
	I am a bright, quick-witted boy/girl	em12 **	—			
	I feel inspired	em14	0.77			
	I am a determined boy/girl	em16	0.793			
	I am an attentive, conscientious person	em17	0.797			
	I am an active boy/girl	em19	0.775			
Negative Emotional Experience (NEX)	I feel tense, overwhelmed, stressed	em02	0.664	0.841	0.884	0.561
	I feel disgusted or upset	em04 **	—			
	I feel guilty	em06 **	—			
	I am a timid boy/girl	em07	0.814			
	I am angry or furious	em08	0.702			
	I am in a bad mood (I get upset or irritated)	em11	0.711			
	I am shy	em13 **	—			
	I feel nervous	em15 **	—			
	I feel afraid	em18	0.754			
	I feel physically restless or worried	em20	0.834			
Academic Performance (AP)	Average grade in Communication and Mathematics	lp	1	—	—	—

Note. * Reverse-coded items, recoded prior to analysis. ** Items removed from the final model. *CR* = composite reliability (rho_c); *AVE* = average variance extracted.

**Table 3:** Discriminant Validity According to the HTMT Criterion.

Variables	1	2	3	4
1. Positive emotional experience	—			
2. Negative emotional experience	0.134	—		
3. Self-esteem	0.447	0.53	—	
4. Academic performance	0.327	0.057	0.245	—

**Table 4:** Structural Model Evaluation: Direct Effects (*N* = 325).

Hypothesis	Path	*β*	*p*	*f* ^2^	Evaluation
H1b	PEX → SELF	0.434	<0.001	0.317	Supported
H2b	NEX → SELF	−0.489	<0.001	0.403	Supported
H1a	PEX → AP	0.272	<0.001	0.063	Supported
H2a	NEX → AP	−0.005	*ns*	0	Rejected
H3	SELF → AP	0.114	0.043	0.009	Supported

Note. PEX = positive emotional experience; NEX = negative emotional experience; SELF = self-esteem; AP = academic performance; *β* = standardized path coefficient; *f*^2^ = effect size; *ns* = non-significant. *R*^2^ of endogenous variables: SELF = 0.408; AP = 0.113.

**Table 5:** Evaluation of the Mediating Effect on Academic Performance.

Total Effect	*β* Total	*p*	Direct Effect	*β* Direct	*p*	Indirect Effect	*β* Indirect	*p*	95% CI	Significant	*VAF* (%)
PEX → AP	0.321	<0.001	H1a (+)	0.272	<0.001	H4 (+)	0.049	0.027	[0.009, 0.093]	Yes	15.27
NEX → AP	−0.06	0.132	H2a (−)	−0.005	*ns*	H5 (−)	−0.056	0.026	[−0.105, −0.011]	Yes	93.33

Note. Bootstrapping with 10,000 subsamples. CI = percentile confidence interval; *VAF* = variance accounted for; *ns* = non-significant; PEX = positive emotional experience; NEX = negative emotional experience; AP = academic performance.

**Table 6:** Evaluation of Measurement Invariance Using MICOM.

Measurement Model	Configural Invariance	Original Correlation	5% Percentile	Compositional Invariance	Mean Difference	Confidence Interval	Equality of Means	Variance Difference	Confidence Interval	Equality of Variances	Total Invariance
PEX	Established	0.999	0.994	Established	−0.2	[−0.22, 0.218]	Yes	−0.02	[−0.31, 0.279]	Yes	No
NEX	Established	0.99	0.988	Established	0.316	[−0.22, 0.218]	No	−0.705	[−0.41, 0.393]	No	No
SELF	Established	0.997	0.995	Established	−0.529	[−0.22, 0.218]	No	−0.423	[−0.36, 0.336]	No	No
AP	Established	1	1	Established	−0.271	[−0.22, 0.215]	No	−0.122	[−0.30, 0.289]	Yes	No

Note. PEX = positive emotional experience; NEX = negative emotional experience; SELF = self-esteem; AP = academic performance.

**Table 7:** Multigroup Analysis (PLS-MGA) of Structural Coefficients.

Hypothesis	Path	*β* G1 (9–12 Years)	*β* G2 (13–17 Years)	Moderation Hypothesis	Δ*β*	*p* (Permutation)	Support
H1b	PEX → SELF	0.357	0.487	H6c	−0.13	0.18	No
H1a	PEX → AP	0.22	0.32	H6a	−0.1	0.355	No
H2b	NEX → SELF	−0.366	−0.549	H6d	0.183	0.035	Yes
H2a	NEX → AP	−0.108	0.032	H6b	−0.14	0.275	No
H3	SELF → AP	0.157	0.036	H6e	0.121	0.312	No

Note. PEX = positive emotional experience; NEX = negative emotional experience; SELF = self-esteem; AP = academic performance.

## Data Availability

The data supporting the findings of this study are not publicly available due to privacy and ethical restrictions, as the participants were minors. Anonymized data may be made available by the corresponding author upon reasonable request, subject to approval by the research team and in accordance with the ethical conditions under which the study was approved.
